# *Quillaja brasiliensis* nanoparticle adjuvant formulation improves the efficacy of an inactivated trivalent influenza vaccine in mice

**DOI:** 10.3389/fimmu.2023.1163858

**Published:** 2023-05-01

**Authors:** Fernando Silveira, Mariana Rivera-Patron, Nikita Deshpande, Soledad Sienra, Jackeline Checa, María Moreno, Jose A. Chabalgoity, Samuel P. Cibulski, Mariana Baz

**Affiliations:** ^1^ Departamento de Desarrollo Biotecnológico, Instituto de Higiene, Facultad de Medicina, Universidad de la República, Montevideo, Uruguay; ^2^ World Health Organization Collaborating Centre for Reference and Research on Influenza at the Peter Doherty Institute for Infection and Immunity, Melbourne, VIC, Australia; ^3^ Unidad de Biología Parasitaria, Facultad de Ciencias, Instituto de Higiene, Facultad de Medicina, Universidad de la República, Montevideo, Uruguay; ^4^ Centro de Biotecnologia – CBiotec, Laboratório de Biotecnologia Celular e Molecular, Universidade Federal da Paraíba, João Pessoa, Paraíba, Brazil; ^5^ Department of Microbiology and Immunology, University of Melbourne at the Peter Doherty Institute for Infection and Immunity, Melbourne, VIC, Australia; ^6^ Department of Microbiology, Infectious Disease and Immunology, Faculty of Medicine, Université Laval, Quebec City, QC, Canada

**Keywords:** *Quillaja brasiliensis* saponins, adjuvant, ISCOM-matrices, protection, influenza virus, intranasal route, neutralizing antibodies

## Abstract

The threat of viral influenza infections has sparked research efforts to develop vaccines that can induce broadly protective immunity with safe adjuvants that trigger robust immune responses. Here, we demonstrate that subcutaneous or intranasal delivery of a seasonal trivalent influenza vaccine (TIV) adjuvanted with the *Quillaja brasiliensis* saponin-based nanoparticle (IMXQB) increases the potency of TIV. The adjuvanted vaccine (TIV-IMXQB) elicited high levels of IgG2a and IgG1 antibodies with virus-neutralizing capacity and improved serum hemagglutination inhibition titers. The cellular immune response induced by TIV-IMXQB suggests the presence of a mixed Th1/Th2 cytokine profile, antibody-secreting cells (ASCs) skewed toward an IgG2a phenotype, a positive delayed-type hypersensitivity (DTH) response, and effector CD4^+^ and CD8^+^ T cells. After challenge, viral titers in the lungs were significantly lower in animals receiving TIV-IMXQB than in those inoculated with TIV alone. Most notably, mice vaccinated intranasally with TIV-IMXQB and challenged with a lethal dose of influenza virus were fully protected against weight loss and lung virus replication, with no mortality, whereas, among animals vaccinated with TIV alone, the mortality rate was 75%. These findings demonstrate that TIV-IMXQB improved the immune responses to TIV, and, unlike the commercial vaccine, conferred full protection against influenza challenge.

## Highlights

- Mono-distributed ISCOM-like nanoparticles (IMXQB) were prepared using a purified fraction of saponins from *Quillaja brasiliensis* (QB) combined with cholesterol and phospholipids under controlled conditions.
- Subcutaneous and intranasal administration of seasonal trivalent inactivated influenza vaccine (TIV) formulated with IMXQB (TIV-IMXQB) elicited high anti-TIV IgG titers and increased hemagglutination inhibition titers and virus-neutralizing capacity compared with TIV alone.
- Intranasal delivery of the adjuvanted vaccine provided complete protection against challenge with A/Uruguay/897/2018 (H1N1)pdm09-like virus.

## 1 Introduction

Prophylactic vaccines are the most successful and cost-effective intervention available to counteract the devasting effects of epidemics and pandemics that arise from infectious diseases. During the current coronavirus disease 2019 (COVID-19) pandemic, candidate vaccines were produced using existing safe and effective platforms that were designed for developing vaccines against other pathogens (1).

Influenza virus infections are also of major concern for global public health, as annual epidemics and sporadic pandemics result in a huge morbidity and mortality burden. Epidemics cause three to five million cases of severe illness and about 290,000 to 650,000 deaths worldwide annually. Current influenza vaccines mainly rely on hemagglutinin (HA) proteins as antigens to induce neutralizing antibodies that can inhibit virus infection and replication in humans (2). These vaccines confer only limited protection due to waning antibodies or the antigenic drift and shift of major influenza surface antigens (3).

The current trivalent and quadrivalent influenza vaccines (TIV and QIV, respectively) are not adjuvanted and confer limited protection, especially in young children and the elderly (2). Two strategies have been used to increase influenza vaccine efficacy; one is to increase the concentration of the antigen in the preparation (high-dose TIV or QIV, which contains four times the antigen of a standard dose of influenza vaccine) (4), and the other is the addition of adjuvants (2). Adjuvants are substances added to vaccines that induce and enhance the magnitude and durability of the immune response. Few adjuvants, including aluminum salts (alum), MF59, and AS03, have been included as adjuvants in influenza vaccines. Alum is well known to trigger robust humoral immune responses that are skewed toward a T helper cell type 2 (Th2) cell response; however, it elicits a weak cellular immune response (5). MF59 and AS03 adjuvants are more effective in eliciting a mixed T helper cell type 1 (Th1) and Th2 response (2, 6). Effective vaccines against intracellular pathogens require stimulation of both humoral and cellular immunity for clearance of infected cells (5).

Saponin-based adjuvants (SBAs) have the ability to promote the effective Th1-biased immune responses that are necessary to overcome intracellular pathogens; therefore, they have been proposed as an alternative to classical adjuvants for the design of new vaccines (7). In particular, saponins from plants of the Quillajaceae family have been proven to be effective adjuvants in several vaccines, especially vaccines against intracellular pathogens, such as *Plasmodium* (the causative agent of malaria) or herpes zoster virus (8). These adjuvants trigger highly antigen-specific antibody responses including the production of a mixture of IgG2a and IgG1, stimulation of the population of cytotoxic T lymphocytes (CTLs), and induction of Th1 cytokines (interleukin (IL)-2 and interferon (IFN)-γ) (8–13).

A major drawback of the use of saponins as adjuvants is their intrinsic toxicity. This unwanted side-effect can be reduced or abrogated by using lipid-based preparations such as adjuvant systems (AS01 saponins in a liposomal formulation) (14) or immunostimulating complexes (ISCOMs and ISCOM-matrices) (11, 15, 16). ISCOMs consist of a 40-nm cage-like, self-assembled, physically and chemically stable nanoparticle combining *Quillaja saponaria* saponins along with cholesterol, phospholipids, and antigens (11, 15, 17). ISCOM-matrices, ISCOM-like preparations without antigens, have been studied and used as vaccine adjuvants in more than 40 clinical trials (18). These studies indicate that vaccines formulated with ISCOM-matrices are safe and well tolerated in humans, with no serious adverse events or clinically significant laboratory abnormalities (18, 19).

Over the last decade, our research team has been studying saponins extracted from the leaves of *Quillaja brasiliensis* (QB) as an alternative to saponins extracted from *Quillaja saponaria* tree bark (9, 20–23). Comprehensive chemical characterization has shown that the two species share a myriad of saponin structures, including well-known saponins with high immunoadjuvant activity, as well as QS-21 (12, 21, 24), a potent adjuvant isolated from the *Q. saponaria* tree and which is currently used in a few licensed vaccines (8, 10, 25). Furthermore, several studies have shown the immunoadjuvant potential of saponins from QB and their ability to form ISCOM-like nanostructures (9, 11, 21, 22, 26). Thus, leaves of QB are a readily renewable source of these important secondary metabolites of high added value and of relevant importance for public health (9, 26).

To better understand the activity of QB ISCOM-matrices as immunoadjuvants, we formulated nanoparticle cage-like structures that were obtained by combining cholesterol, phospholipids, and QB saponin fractions, called IMXQB, as previously described (16). In this study, we used a seasonal inactivated TIV and IMXQB nanoparticles as an adjuvant. The IMXQB nanoparticle influenza vaccine (TIV-IMXQB) elicited robust neutralizing antibody titers against the challenge virus (A/Uruguay/897/2018 (H1N1)pdm09-like), generated mixed Th1/Th2 cytokine profiles, and conferred full protection in the lower respiratory tract of mice when administered by either the subcutaneous or the intranasal route.

## 2 Materials and methods

### 2.1 ISCOM-matrices QB-based adjuvant: preparation and characterization

QB (A. St.-Hil. et Tul) Mart. leaves were collected in Parque Battle, Montevideo, Uruguay (–34.89302, –56.15727) (voucher MVFQ 4321, deposited at the Herbarium of the Facultad de Química, Universidad de la República). Extraction and purification of saponins fractions were carried out as previously described (27).

IMXQB was prepared using the dialysis method previously described (16), and ISCOM-matrices were assembled using Quil A^®^ (IMXQA). IMXQA and IMXQB were formulated in parallel. The nanoparticles obtained were sterilized by filtration using a 0.22-μm syringe filter and maintained at 4°C. The endotoxin level in IMXQB was analyzed using the Amebocyte Lysate assay kit (Pierce^TM^ Chromogenic Endotoxin Quan Kit, Thermo Scientific, China). The endotoxin level in the IMXQB (3 mg/mL stock solution) was less than 0.5 EU (endotoxin units)/mL, which is in compliance with the Food and Drug Administration guidance.

The hydrodynamic diameter of IMXQB nanoparticle formulations was determined using the dynamic light scattering (DLS) technique (Zetasizer Nano ZS, Malvern Instruments, Ltd.), and the preparation was visualized by transmission electron microscopy (TEM) with a JEMM 2100 (JEOL, Japan) high-resolution transmission electron microscope using a previously described methodology (16).

### 2.2 Toxicity assays

The hemolytic activity of saponins is a major indicator of cytotoxicity. The hemolytic activity of QB and Quil A^®^ saponins as well as of IMXQB and IMXQA nanoparticles was tested over a range of concentrations (5–150 µg/mL), as previously described (26, 28). Saline solution and *Q. saponaria* saponins (Sigma Aldrich, USA) at 250 µg/mL were used as indicators of 0% and 100% hemolysis, respectively. The hemolytic activity was expressed as the endpoint concentration capable of inducing hemolysis in 50% (HD_50_) of red blood cells (RBCs).

Cytotoxicity in the Madin–Darby canine kidney cell line (MDCK; ATCC CCL-81) was determined by MTT (3-(4,5-dimethylthiazol-2-yl)-2,5-diphenyl-2H-tetrazolium bromide) assay. Briefly, cells were seeded in Dulbecco’s modified Eagle medium (DMEM; Capricorn Scientific) supplemented with 10% fetal bovine serum (FBS; Capricorn Scientific) and antibiotics (penicillin 100 IU/mL, streptomycin 100 g/mL), added to 96-well cell culture plates (Greiner Bio-One, Darmstadt, Germany) at 4.0 × 10^4^ cells/well, and incubated at 37°C in a humid atmosphere with 5% CO_2_. After 18 hours, the medium was removed and 100 µL of the culture medium containing different concentrations of SBAs (10 to 700 µg/mL) was added to each well in triplicate. The plates were incubated as above. After 24 hours, 50 µL of 2 mg/mL MTT (Sigma Aldrich, USA) was added to each well and the cells were incubated for a further 4 hours; the optical density was measured in a microplate reader at 570 nm (11, 26).

### 2.3 Nanoparticle adjuvant vaccine formulation and mice immunization

Antigens were obtained from the 2019 trivalent split-inactivated influenza virus vaccine (VAXIGRIP, Sanofi-Pasteur), which included A/California/2570/2019 (H1N1)pdm09, A/Hong Kong/2671/2019 (H3N2), and B/Washington/02/2019 containing 15 µg of HA per strain in 0.5 mL. A dose of 7.5 µg (2.5 µg of each HA) was used in each immunization, except for intranasal immunization, in which case 3.75 µg/dose was used. The experimental vaccines were prepared under aseptic conditions, filtered through 0.22 µm, and kept at 4°C until administration.

BALB/c mice were purchased from Dirección de Laboratorios Veterinarios (Ministerio General de Agricultura y Pesca, Uruguay), while the CD1 mice were produced at the Instituto de Higiene (Universidad de la República). The animals were 8–10 weeks old at the time of the experiment. All procedures were carried out in strict accordance with “Comisión Honoraria de Experimentación Animal” (CHEA-Universidad de la República) guidelines and were approved by the Uruguayan University Research Ethics Committee (approval number 070153-000310-17). Animals were appropriately housed at a controlled temperature (22 ± 2°C) and humidity (50–60%) with a 12/12 hour light/dark cycle and with access to food and water *ad libitum*.

Female BALB/c and CD1 mice were immunized on days 0 and 14 with the IMXQB nanoparticle influenza vaccine through the subcutaneous (s.c.) or intranasal (i.n.) route. BALB/c female mice were divided into five groups (*n* = 10 per group) and CD1 female mice were divided into three groups (*n* = 5 per group). In the case of s.c. immunizations, animals were injected in the hind neck with 100 µL of TIV as antigen (7.5 µg/dose) plus 100 µL of saline (non-adjuvanted group), IMXQB (5.0 µg/dose), or IMXQA (5.0 µg/dose). Nanoparticles without antigens (IMXQB, 5.0 µg/dose) and saline-treated animals were included as controls. For i.n. immunization, mice (*n* = 8) were anesthetized with ketamine–xylazine and received half the dose used for s.c. immunization: 3.75 µg/dose of TIV plus 50 µL of saline quantitat suficient per (q.s.p) (non-adjuvanted group) or 3.75 µg/dose of TIV adjuvanted with IMXQB (2.5 µg).

Blood samples were collected immediately prior to each immunization (days 0 and 14) and challenge (day 28). Sera were stored at −20°C until use. The concentration of nanoparticles (5.0 µg/dose) used in this study was defined as saponin concentration in nanoparticle adjuvant.

### 2.4 Enzyme-linked immunosorbent assay

Anti-TIV IgM, IgG, and anti-isotypes IgG1, IgG2b, IgG2c, and IgG3 were determined by indirect ELISA as described previously (9). A standard curve was built using a pool of sera from immunized groups, and antibody titers were expressed in arbitrary units per mL (AU/mL). IgM titers were expressed in optical density (OD) because they were much lower than those of the other isotypes. For the determination of IgM titers, all samples were diluted 1/500.

### 2.5 Hemagglutination inhibition and microneutralization assays

Antibody titers against influenza A/California/07/2009 (H1N1)pdm09-like virus in mouse serum samples were measured by a hemagglutination inhibition (HAI) assay in accordance with standard protocols (29) and by a microneutralization (MN) assay as previously described (30).

For HAI, non-specific inhibitors were removed from the serum by overnight treatment with receptor-destroying enzymes (Denka Seiken, Tokyo, Japan). Physiologic saline solution was then added to achieve a 1:10 dilution. A/California/07/2009 (H1N1)pdm09-like virus (4 HA units in 25 µL) was added to an equal volume of treated serum previously diluted in a microtiter plate. After 30 minutes of incubation at room temperature, 50 µL of a 0.5% turkey red blood cell (TRBC) solution was added to the mixture, and then the mixture was incubated for 45 minutes before evaluation of hemagglutination. The HAI titer was recorded as the reciprocal of the highest dilution of serum at which hemagglutination was inhibited.

For the MN assay, sera were first inactivated at 56°C, and serial twofold dilutions were prepared, starting at 1:20 dilution. Equal volumes of serum and A/California/07/2009 (H1N1)pdm09-like virus were mixed and incubated for 60 minutes at room temperature. The residual infectivity of the virus–serum mixture was determined in MDCK cells using four wells for each serum dilution. Neutralizing antibody titer was defined as the reciprocal of the serum dilution that completely neutralized the infectivity of 100 TCID_50_ (median tissue culture infectious dose) of A/California/07/2009 (H1N1)pdm09-like as determined by the absence of a cytopathic effect (CPE) on MDCK cells at day 4 and calculated using the Reed–Muench method (30).

### 2.6 Splenocyte cell culture and cytokine determination

Spleens were collected from female CD1 mice 14 days after the second immunization under aseptic conditions, immersed in RPMI 1640 medium (Gibco), minced, and mechanically dissociated to obtain a homogeneous cell suspension. Erythrocytes were lysed with an ACK (ammonium-chloride-potassium) lysis buffer. After centrifugation (380 × g at 4°C for 10 minutes), pelleted cells were washed three times in RPMI 1640 and resuspended in the same medium supplemented with 0.05 mM 2-mercaptoethanol, 100 IU/mL penicillin, 100 μg/mL streptomycin, 2 mM L-glutamine, and 10% FBS (RPMI complete medium), and passed through a 100-µm cell strainer (BD Falcon). Cell counting by trypan blue dye exclusion revealed > 95% viability.

For the flow cytometry analysis, splenocytes were incubated with antibodies for 30 minutes at 4°C (5 × 10^5^ cells, 100 µL/well). Splenocytes were stained with the following surface-staining antibodies: CD4-APC-Cy7 (BD Biosciences; clone RM4.5), CD8-PerCP (BD Biosciences; clone 53-6.7), CD62L-APC (BD Biosciences; MEL-14), and CD127-PE-Cy7 (BioLegend, San Diego, CA, USA; clone A7R34). All staining procedures were conducted on ice, and reagents were purchased from Life Technologies. Cell populations were analyzed using a FACS Canto II flow cytometer (BD Biosciences). Retrieved data were analyzed by FACS Diva software (version 6.1.3; BD Biosciences).

The splenocyte proliferation assay was performed as described elsewhere (11, 31). Cells were seeded in a flat-bottom microtiter plate (2.5 × 106 cells per well) (Greiner Bio-One) and pulsed for 3 days with TIV antigen (0.5 µg/mL) and concanavalin A (5 µg/mL), or RPMI complete medium only was added and the supernatants harvested. Plates were then incubated at 37°C in a humid atmosphere with 5% CO_2_. After 68 hours, 50 μL of MTT (Sigma) solution (2 mg/mL) was added to each well and the plates were incubated for 4 hours. The plates were centrifuged at 1,400 × g for 5 minutes and the untransformed MTT was removed carefully by pipetting. Next, 100 μL of dimethyl sulfoxide (DMSO) solution (92 μL of DMSO with 8 μL of 1 N HCl) was added to each well. After 15 minutes of incubation, the absorbance was measured in an ELISA reader at 570 nm. The stimulation index (SI) was calculated as the absorbance ratio of mitogen-stimulated cultures and non-mitogen-stimulated cultures.

The concentrations of IFN-γ, tumor necrosis factor (TNF)-α, IL-10, IL-4, and IL-5 were determined by a commercial capture ELISA kit in the supernatant (BioLegend). Concentrations of cytokines were calculated from interpolation of the cytokine standard curve.

### 2.8 ELISpot assay for TIV-specific antibody-secreting cells and DTH assay

The frequency of TIV-specific antibody-secreting cells (ASCs) producing IgG, IgG1, or IgG2 isotypes were determined on day 30 post priming in BALB/c mice (*n* = 5) using the ELISpot assay, as previously described by our group (9). Results are expressed as the number of spot-forming units per 2 × 10^5^ cells (splenocytes) for anti-influenza ASC IgG, IgG1, and IgG2a.

DTH responses were tested 28 days post priming in BALB/c mice. Briefly, mice (*n* = 5) were intradermally injected with 1 µg of TIV in one footpad of the hind limb. The thickness of the injected footpads was measured 24 hours later with a caliper. Swelling in mice inoculated with saline was used as a basal condition. TIV-specific DTH responses in each animal were determined as the difference between the thickness of the injected footpad and the average footpad thickness in animals injected with saline (9, 11, 12).

### 2.9 Mouse challenge

The influenza virus used for the challenge (A/Uruguay/897/2018 (H1N1)pdm09-like virus) was isolated from a nasal swab taken from a female patient at the Reference Center for Influenza and other Respiratory Viruses, National Institutes of Health Laboratories (DLSP-MSP), Montevideo, Uruguay. The isolate was propagated in MDCK cells and the virus working stock was stored at –80°C until use.

Thirty days after the priming, all animals in each group (*n* =10 per group) were anesthetized with ketamine–xylazine and intranasally infected with 1 × 10^6^ TCID_50_ iA/Uruguay/897/2018 (H1N1)pdm09-like virus in 50 μL of saline solution. On day 5 post infection, five mice from each group were euthanized, and their lungs were harvested, flash frozen, and kept at –80°C until viral titer determination. Lungs were homogenized in 1 mL of phosphate-buffered saline (PBS), clarified by centrifugation at 2,500 rpm for 10 minutes, and titered in 24- and 96-well plates containing MDCK monolayers. Viral titers were determined by TCID_50_ in MDCK cells using the Reed–Muench method (32, 33). The remaining five mice per group were observed for 14 days post infection and monitored daily for weight loss and other clinical signs (ruffled fur and lethargy). Animals were euthanized if weight loss exceeded 20% of initial body weight.

### 2.10 Statistical analysis

Statistical significance was assessed by a one-way-ANOVA Kruskal–Wallis test with an uncorrected Dunn’s *post hoc* test correction for multiple comparisons compared with the control group (unadjuvanted TIV antigen). The Mann–Whitney *U*-test was used to compare the two groups. GraphPad Prism version 7.00 (GraphPad Software, Inc.) was used for data analysis.

## 3 Results

### 3.1 IMXQB nanoparticle characterization and toxicity profile

**Figure 1:**
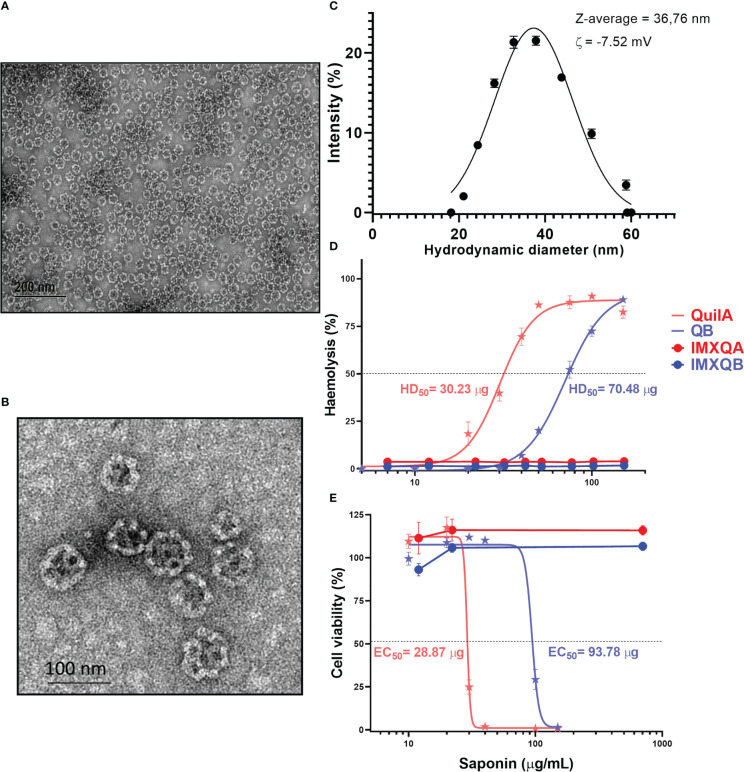
IMXQB nanoparticle characterization. **(A)** Transmission electron microscopy (TEM) image of an IMXQB nanoparticle. **(B)** High-magnification IMXQB TEM image. Images were obtained after negative staining with uranyl acetate. **(C)** IMXQB size distribution determined by dynamic light scattering (DLS) measurements. **(D)** Hemolytic activity of *Q. brasiliensis* (QB) and Quil A^®^ saponins and their nanoparticulate formulations (IMXQB and IMXQA). **(E)** Madin–Darby canine kidney cell line (MDCK) cytotoxicity of QB or Quil A^®^ saponins and their nanosized formulations (IMXQB and IMXQA). Cell viability was measured by MTT (3-(4,5-dimethylthiazol-2-yl)-2,5-diphenyl-2H-tetrazolium bromide) assay 24 hours after treatment. All results are presented as the mean value ± standard deviation. HD_50_ (hemolytic dose 50%) and IC_50_ (half-maximal inhibitory concentration values) were calculated and plotted in GraphPad Prism 9.

IMXQB was prepared using a purified fraction of saponins from QB combined with cholesterol and phospholipids under controlled conditions (16). High-resolution TEM showed that IMXQB consisted of mono-distributed nanoparticles of approximately 40 nm in diameter that resembled the cage-like structures characteristic of ISCOMs (**Figures 1A, B**). DLS analysis showed that the Z-average diameter for IMXQB was 36.76 ± 0.32 nm. In **Figure 1C**, the size distribution of IMXQB is presented. Measurements of zeta potential (ζ) indicated that IMXQB has an overall negative surface charge of –7.52 ± 1.1 mV. Regarding IMXQA nanoparticles, similar size distribution (47.9 ± 0.38 nm) and ζ potential were observed (–5.43 ± 1.03 mV) (data not shown).

The hemolytic activity, defined as the concentration of saponins able to lyze 50% of rabbit RBCs (HD_50_), was 70.48 and 30.23 µg for QB and Quil A^®^, respectively (**Figure 1D**). However, when formulated as ISCOMs, the hemolytic activity of the IMXQA and IMXQB nanoparticles was abrogated (**Figure 1D**), and the nanoparticles showed no measurable hemolytic activity even at 150 µg/mL. The cytotoxicity of QB and Quil A^®^ in MDCK cells was moderate, with CC_50_ (concentration cytotoxic to 50% of cells) of 93.78 µg/mL and 28.87 µg/mL, respectively, whereas IMXQB and IMXQA showed no measurable toxicity (**Figure 1E**). In addition, no signs of local toxicity (local swelling, loss of hair, and piloerection) were observed in mice inoculated with the vaccine formulated with IMXQB or IMXQA.

### 3.2 Subcutaneous immunization with TIV-IMXQB significantly improved anti-TIV immunity and protected mice against lethal challenge with A/Uruguay/897/2018 (H1N1)pdm09-like virus

**Figure 2:**
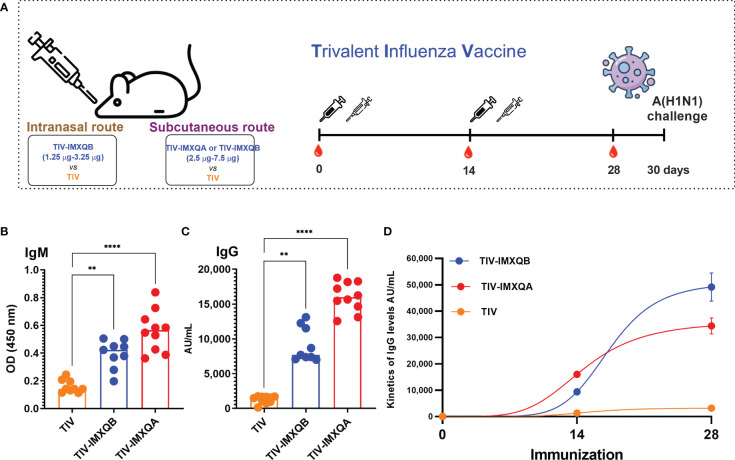
Two doses of subcutaneous (s.c.) injection of an adjuvanted influenza vaccine (TIV-IMXQB) augment the antibody response of commercial trivalent influenza vaccines (TIVs) in mice. **(A)** Schematic vaccination schedule. Female BALB/c mice were vaccinated by the s.c. route on days 0 (priming) and 14 (booster) with either TIV-IMXQB, the TIV-IMXQA-adjuvanted formulation, or TIV alone (commercial vaccine), and antibody levels were measured in sera on day 28. **(B)** Specific anti-TIV IgM levels 14 days post priming. **(C)** IgG antibody levels. **(D)** Total IgG kinetics (days 14 and 28)). The median value is indicated by a line, and the dots indicate individual values. The statistical analyses were performed using Kruskal–Wallis and uncorrected Dunn’s *post hoc* test, comparing every group against the unadjuvanted (TIV alone) group as control. Statistically significant differences are indicated: **p < 0.01 and ****p< 0.0001.

Vaccines containing 7.5 µg/dose of HA with IMXQB (TIV-IMXQB), IMXQA (TIV-IMXQA), or alone were administered subcutaneously. IMXQB nanoparticulate adjuvant (without TIV antigen) and a saline group were included as controls. The presence of specific anti-TIV antibodies was evaluated by ELISA 2 weeks after the first (priming) and second (booster) immunizations. The experimental design is shown in **Figure 2A**. The immune responses promoted in the control groups (saline, IMXQB) were not detected by ELISA (data not shown).

On day 14 post priming, a significant increase in specific IgM antibodies was observed in groups vaccinated with TIV-IMXQB (*p <* 0.01) and TIV-IMXQA (*p <* 0.0001), compared with the TIV group (**Figure 2B**). Similarly, a significant increase in total anti-TIV IgG antibodies was observed in the groups immunized with TIV-IMXQB (*p <* 0.01) or TIV-IMXQA (*p <* 0.0001) compared with the TIV-alone group (**Figure 2C**). As expected, the immune response in all groups was boosted after the second-shot immunization (**Figure 2D**).

**Figure 3:**
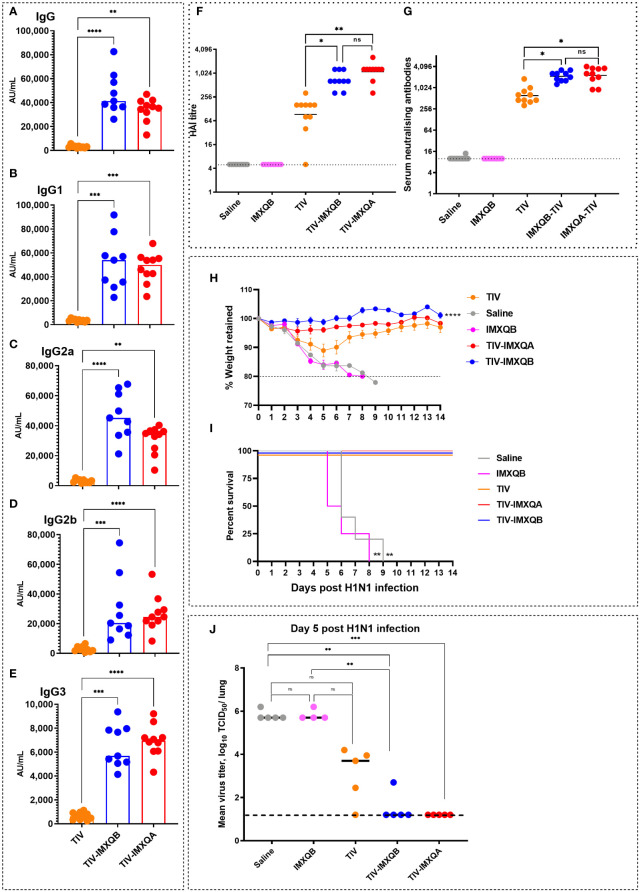
Subcutaneous (s.c.) injections of TIV-IMXQB-adjuvanted trivalent influenza vaccine (TIV) elicited higher antibody levels than commercial TIV alone and conferred nearly complete protection upon challenge. Female BALB/c mice were vaccinated by the s.c. route on days 0 (priming) and 14 (booster) with either TIV-IMXQB, the TIV-IMXQA-adjuvanted formulation, or TIV alone (commercial vaccine). Total anti-TIV IgG **(A)**, IgG1 **(B)**, IgG2b **(C)**, IgG2a **(D)**, and IgG3 **(E)** were determined 2 weeks after the second immunization (day 28). The median value is indicated by a line, and the dots indicate individual values. Anti-influenza virus antibody titers in serum samples were measured by hemagglutination inhibition (HAI) **(F)** or microneutralization (MN) **(G)** assays. The geometric mean value is indicated by a line, and the dots indicate individual values. Thirty days after priming, all animals in each group (*n* = 10 per group) were intranasally challenged with 1 × 10^6^ TCID (median tissue culture infectious dose)/50 µL of A/Uruguay/897/2018 (H1N1)pdm09 influenza virus. Five animals per group were monitored for weight loss **(H)** (represented by mean and error) and mortality **(I)**. The remaining five mice per group were used to determine viral titers in the lungs on day 5 post challenge **(J)**. The median value is indicated by a line, and the dots indicate individual values. Percentage survival compared with trivalent influenza vaccine (TIV) alone (unadjuvanted TIV antigen) was determined by a log-rank (Mantel–Cox) test. Statistical analyses were performed using the non-parametric Kruskal–Wallis test with uncorrected Dunn’s *post hoc* test for multiple comparisons or a log-rank (Mantel-Cox) test, and each group was compared with the TIV mock group. In **(A-G, J)**, median values are indicated by horizontal lines and dots indicate individual values for each animal. The dotted horizontal line in Figure **(J)** represents the lower limit of detection. Significant differences are indicated: *p < 0.05, **p < 0.01, ***p< 0.001, and ****p< 0.0001. ns, non-significantly.

The levels of total anti-TIV IgG and IgG isotypes on day 28 post priming are shown in **Figure 3**. Similar to day 14 post priming, a significant increase in anti-TIV IgG was observed in mice immunized with TIV-IMXQB and TIV-IMXQA when compared with mice immunized with TIV alone (*p <* 0.0001 and *p* < 0.01, respectively). IgG isotypes (IgG1, IgG2a, IgG2b, and IgG3) were also all significantly enhanced in TIV-IMXQB- and TIV-IMXQA-vaccinated groups (*p* < 0.01, in all isotypes). No differences in total IgG or IgG isotype levels were observed when comparing TIV-IMXQB and TIV-IMXQA with TIV alone (**Figures 3A–E**).

To further characterize the antibody response elicited by the adjuvanted nanoparticle formulations against the A/H1N1 influenza virus, we performed HAI and MN assays. Both vaccines containing saponin-based adjuvant (TIV-IMXQB or TIV-IMXQA) elicited significantly higher titers of HAI (geometric mean titer (GMT) 640 and 1,280, respectively) and neutralizing antibodies (GMTs 2,153 and 2,032, respectively) as compared with the TIV alone, which elicited GMTs of 92 and 611 by HAI and MN assays, respectively (*p* < 0.05) (**Figures 3F, G**). A similar profile was observed for CD1 mice (data not shown).

Thirty days after the first immunization, all immunized mice were intranasally challenged with a lethal dose (1 × 10^6^ TCID_50_/50 µL) of A/Uruguay/897/2018 (H1N1)pdm09-like virus and monitored daily for clinical signs of disease, weight loss, and mortality for 14 days. All non-immunized mice rapidly deteriorated and died between days 6 and 9 (**Figures 3H, I**). Significant weight loss was observed in mice in the non-adjuvanted TIV group, with a weight loss of 10% by day 5, but with eventual recovery (**Figure 3H**). In contrast, the TIV-IMXQB and TIV-IMXQA groups did not show significant loss in body weight.

On day 5 post challenge (p.c.), half of the mice in each group (*n* = 5) were euthanized to assess viral load in the lungs. In mock- and IMXQB-immunized mice, the mean titers were 10^5.5^ TCID_50_/lung. Animals that received TIV only exhibited a less than 2-log reduction in virus titer (10^3.7^ TCID_50_/lung), which was not statistically significant when compared with the control group. However, mice receiving TIV-IMXQB were nearly cleared of the challenge virus: only one animal out of five had a very low but detectable virus (10^2.7^ TCID_50_/lung) and those receiving TIV-IMXQA were fully protected (**Figure 3J**).

### 3.3 TIV-IMXQB induces a Th1/Th2 mixed immune response

**Figure 4:**
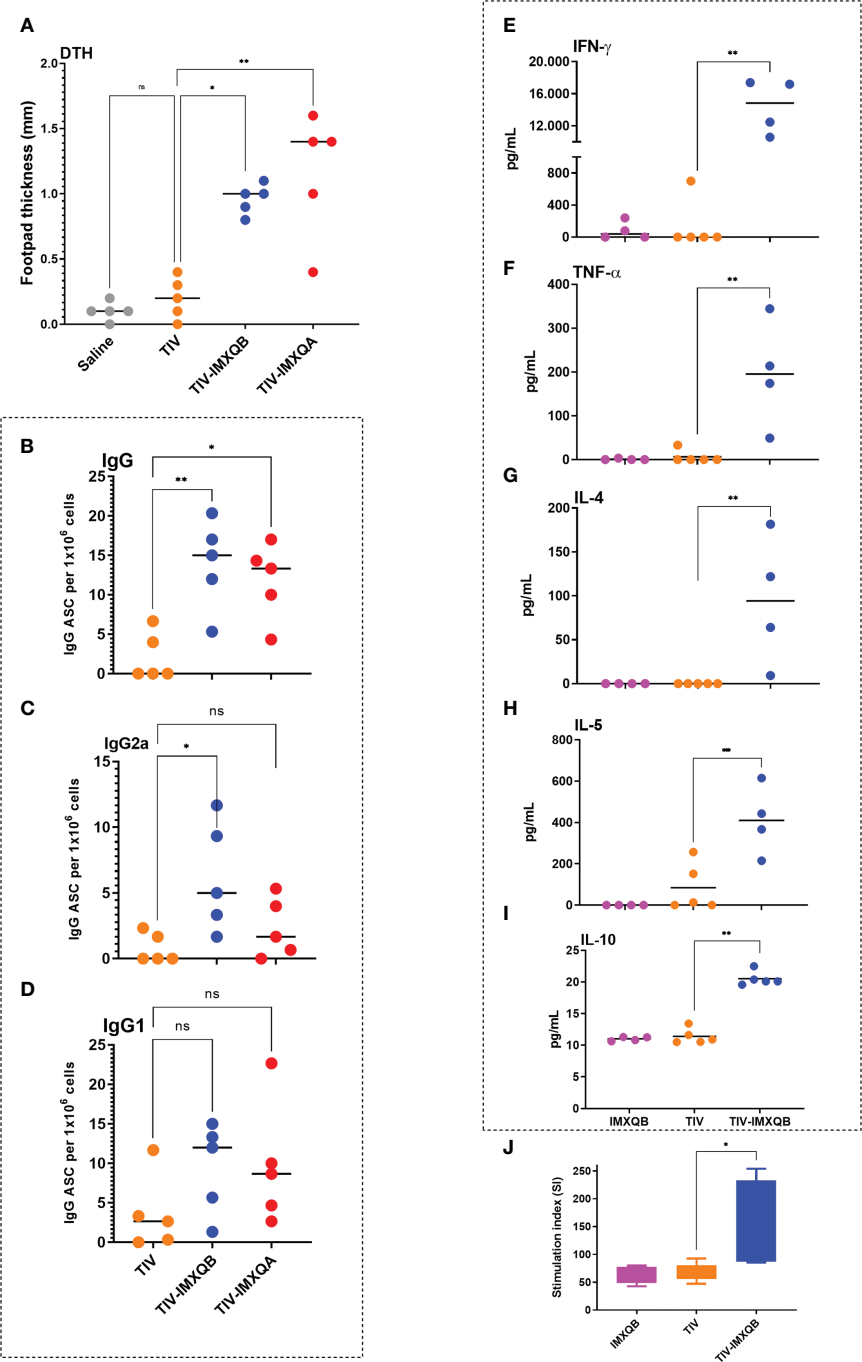
TIV-IMXQB promotes T helper cell type 1 (Th1)/T helper cell type 2 (Th2) mixed immune responses. Female BALB/c mice (*n* = 5) were inoculated subcutaneously on days 0 (priming) and 14 (booster) with saline, trivalent influenza vaccine (TIV) alone, TIV-IMXQB, or TIV-IMXQA. The delayed-type hypersensitivity (DTH) controls were mice injected with TIV alone. The DTH **(A)** and ELISpot **(B–D)** assays were carried out 2 weeks after the second immunization. The median value is indicated by a line, and the dots indicate individual values. CD1 female mice (*n* = 5) were inoculated subcutaneously on days 0 (priming) and 14 (booster) with either the control formulation (IMXQB or TIV alone) or TIV-IMXQB. The cytokine evaluation **(E–I)** and splenocyte proliferation assay (represented as stimulation index, SI) **(J)** were carried out 2 weeks after the second immunization. Spleens were harvested from immunized female CD1 mice and cultured with TIV antigen for 3 days. Cytokines in the supernatant were quantified by capture with ELISA **(E–I)** The median value is indicated by a line, and dots indicate individual values. Splenocytes from each mouse were prepared and pulsed *in vitro* with TIV for 4 days and proliferation was measured by an MTT assay, and these results are shown as box-and-whisker plots. Statistical analyses were performed in all cases using a Kruskal–Wallis test with uncorrected Dunn’s *post hoc* test for multiple comparisons. Significant differences compared with the unadjuvanted vaccine (TIV) are indicated: *p < 0.05) and **p< 0.01. ns, non-significantly.

The TIV-specific DTH reaction response was measured 2 weeks after boosting. A significant DTH response was observed in mice immunized with TIV-IMXQB and TIV-IMXQA compared with those immunized with TIV alone (*p <* 0.05 and *p <*0.01, respectively) (**Figure 4A**).

The frequency of TIV-specific ASCs producing IgG and IgG1 or IgG2 isotypes was assessed by ELISpot. As shown in **Figure 4B**, higher anti-TIV ASC IgG numbers were found in animals immunized with TIV-IMXQB (*p* < 0.01) or TIV-IMXQA (*p <* 0.05) than in those immunized with non-adjuvanted TIV. Immunization with TIV-IMXQB promoted the generation of more anti-TIV IgG2a-ASCs than immunization with TIV alone (*p <* 0.05, **Figure 4C**). Specific ASCs producing IgG1 were also detected in the group receiving nanoparticles as adjuvant and the group receiving TIV alone, but no statistically significant differences between groups were observed (**Figure 4D**).

The cellular immune response evoked was investigated by *in vitro* cytokine release in splenocytes after antigen restimulation. Immunization with TIV-IMXQB resulted in a different pattern of cytokine production from the one obtained using the non-adjuvanted vaccine. As shown in **Figures 4E, F**, a significant increase in Th-related cytokines (INF-γ and TNF; *p* < 0.01) was observed in splenocytes from TIV-IMXQB-immunized animals compared with mice immunized with TIV alone. In addition, TIV-IMXQB promoted an increase in Th2 cytokines (IL-4 and IL-5; *p <* 0.01 and *p <*0.05, respectively) (**Figures 4G, H**) and triggered a significant production of IL-10 (**Figure 4I**). No differences were observed in the production of cytokines between animals vaccinated with TIV or adjuvant only.

A splenocyte proliferation assay was performed after antigen stimulation. Significant cell proliferation was observed in mice immunized with TIV-IMXQB compared with those immunized with TIV alone (**Figure 4J**; *p* < 0.05). Splenocyte proliferation rates were similar in the group vaccinated with TIV and the group vaccinated with IMXQB alone (**Figure 4J**).

### 3.4 Immunization with TIV-IMXQB induces recruitment of more CD4^+^ and CD8^+^ effector T cells in spleen

**Figure 5:**
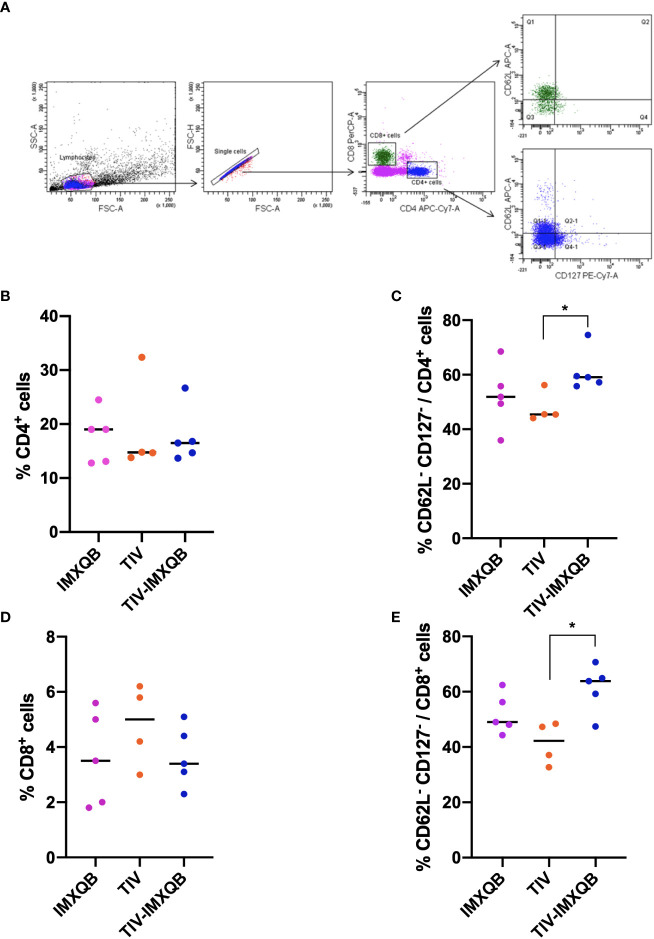
TIV-IMXQB adjuvanted influenza vaccine recruits more effector-T cells than the unadjuvanted vaccine. CD1 female mice (n=5) were inoculated subcutaneously on days 0 (priming) and 14 (booster) with either the control formulation (IMXQB or trivalent influenza vaccine (TIV) alone) or TIV-IMXQB. Recruitment of effector T cells (CD62L^-^CD127^-^) was assessed by flow cytometry. Splenocytes obtained on day 28 post primed were stained with the following surface-staining antibodies: CD4-APC-Cy7, CD8-PerCP, CD62L-APC, and CD127-PE-Cy7. **(A)** Gating strategy was performed. No differences were found in the recruitment of CD4^+^ **(B)** or CD8^+^ **(D)** T cells among groups. TIV-IMXQB formulation was able to promote higher recruitment of CD62L^-^CD127^-^ cells within CD4^+^ **(C)** and CD8^+^ **(E)** T cells than the unadjuvanted formulation (TIV). The median value is indicated by a line, and the dots indicate individual values. Statistical analyses were performed by a Mann–Whitney *U*-test (*t*-test non-parametric) test comparing every group against the others. Statistically significant differences are indicated with stars: *(P < 0.05) and horizontal line.

Vaccination did not induce changes in the percentage of helper and cytotoxic T-cell populations in spleens (**Figures 5A, B, D**). However, TIV-IMXQB promoted an augmentation of effector phenotypes within the above-mentioned cell populations. We found a higher percentage of effector CD4^+^ and CD8^+^ T cells (determined to be CD62^–^ CD127^–^) in the spleen of TIV-IMXQB-vaccinated animals than in the spleen of those vaccinated with TIV alone (**Figures 5C, E**; *p <* 0.05). Of note, the specificity of these effector cells has been not determined. No differences between TIV-alone and IMXQB-alone treatments were found in any of the cell populations studied.

### 3.5 Intranasal immunization with TIV-IMXQB confers strong protection against virus challenge

**Figure 6:**
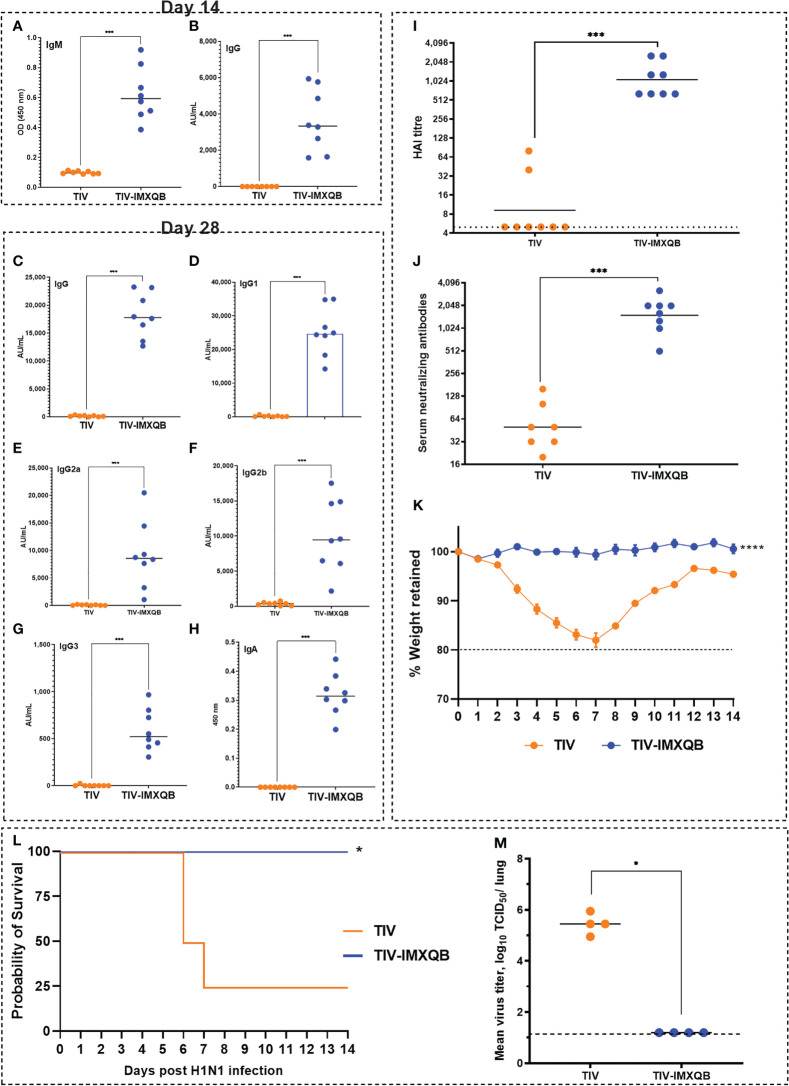
Intranasal delivery of TIV-IMXQB elicited a robust antibody response and promote protection against A/Uruguay/897/2018 (H1N1)pdm09-like virus challenge. Female BALB/c mice were vaccinated intranasally on day 0 (priming) and 14 (booster) with either trivalent influenza vaccine (TIV) alone (commercial vaccine) or TIV-IMXQB and antibody levels were measured in sera. Anti-TIV IgM **(A)** and IgG **(B)** titers 2 weeks post-priming (day 14) were measured. Total anti-TIV IgG **(C)**, IgG1 **(D)**, IgG2a **(E)**, IgG2b **(F)**, IgG3 **(G)**, and IgA **(H)** were determined 2 weeks after the second immunization (day 28). The median value is indicated by a line, and the dots indicate individual values. Anti-influenza virus antibody titers in serum samples were measured by hemagglutination inhibition (HAI) **(I)** or by microneutralization (MN) **(J)** assays. The geometric mean value is indicated by a line, and the dots indicate individual values. Thirty days after the priming all animals in each group (N=8/group) were intranasally challenged with 1 × 10^6^ tissue culture infectious dose (TCID)_50_/50µL of A/Uruguay/897/2018 (H1N1)pdm09-like virus. Four animals per group were monitored for weight loss **(K)** (represented by mean and error) and mortality **(L)**. Viral titers in the lungs were determined on day 5 post challenge (N=4/group) **(M)**. The median value is indicated by a line, and the dots indicate individual values. Statistical analyses were performed using the nonparametric Kruskal–Wallis test with uncorrected Dunn’s post-test for multiple comparisons. Probability of survival as determined by Log-rank (Mantel-Cox) test compared to TIV alone. **(A-K, M)** median values are indicated by horizontal lines and dots indicate individual values for each animal. The dotted horizontal line in Figure **(M)** represents the lower limit of detection. Significant differences are indicated: *(*P < 0.05*), ***(*P < 0.001*) and ****(P < 0.0001) as determined by Log-rank (Mantel-Cox) test compared to TIV alone.

On day 14 after intranasal immunization, a significant increase in IgM and IgG levels was observed in TIV-IMXQB-vaccinated animals compared with those vaccinated with TIV alone (**Figures 6A, B**; *p* < 0.001). Intranasal delivery of unadjuvanted TIV did not elicit detectable anti-TIV IgG antibodies after booster immunization (day 28 post priming) (**Figure 6C**). However, TIV-IMXQB-immunized mice produced significantly higher (*p* < 0.001) IgG responses than TIV-immunized mice.

The TIV-IMXQB vaccine elicited significantly higher (*p <* 0.001) IgG1 (**Figure 6D**), IgG2a (**Figure 6E**), IgG2b (**Figure 6F**), and IgG3 (**Figure 6G**) titers than TIV alone. In addition, the TIV-IMXQB vaccine, but not TIV, was able to induce anti-TIV serum IgA antibodies 28 days post priming (**Figure 6H**; *p* < 0.001). More remarkably, two doses of TIV-IMXQB induced higher HAI (**Figure 6I**; *p <* 0.001) and neutralizing antibody titers (**Figure 6J**, *p <* 0.001) against A/Uruguay/897/2018 (H1N1)pdm09-like influenza virus (titers ranged from 640 to 2,560 (GMT = 1,076) in the HAI assay and from 508 to 3,225 (GMT = 1,522) in the MN assay) than did TIV alone (titers ranged from 5 to 80 (GMTs = 9) in the HAI assay and from 20 to 160 (GMT = 50) or in the MN assay).

As performed for s.c. administration, 30 days after the first immunization, all intranasally immunized mice were challenged with a lethal dose of A/Uruguay/897/2018 (H1N1)pdm09-like virus. Animals vaccinated with TIV alone had a rapid decrease in body weight (**Figure 6K**), which ended with the death of 75% of animals (**Figure 6L**). In contrast, animals vaccinated with TIV-IMXQB showed no body weight loss, signs of disease, or mortality (**Figures 6K, L**).

A similar pattern to the subcutaneous route was observed when animals were vaccinated intranasally with TIV-IMXQB and challenged with 1 × 10^6^ TCID_50_/50 µL of A/Uruguay/897/2018 (H1N1)pdm09-like virus. Indeed, all animals were fully protected against replication in the lungs (no virus detected). However, the mean virus titer in mice immunized with TIV alone was 10^5.45^ TCID_50_/lung (**Figure 6M**).

## 4 Discussion

Influenza A viruses cause seasonal epidemics and sporadic pandemics associated with high morbidity and mortality. Influenza disease burden, especially in very young children and the elderly, has been substantially reduced by the use of influenza virus vaccines (34). Influenza vaccines are formulated every year to match the circulating strains. Nevertheless, vaccine efficacy is not optimal and can be dramatically low in the case of an antigenic mismatch between the vaccine and the circulating virus strain, conferring suboptimal protection. Therefore, there is an urgent need to improve vaccine formulations to trigger optimal responses against medically important pathogens. The efficacy of a vaccine can be improved by the addition of adjuvants, which usually help to stimulate the immune system in a more effective way (2, 5, 17).

The value of saponins from plants of the Quillajaceae family as adjuvants to significantly boost immune responses has long been demonstrated (7, 10, 20). However, they also show toxicity in mammalian cells. This unwanted effect is eliminated when saponins are formulated in micellar formulations (ISCOMs, ISCOM-matrices, and liposome-based formulations such as AS01). The mixture of saponins with sterols reduces or abrogates the toxicity of these molecules while maintaining, and often increasing, their immunostimulatory properties (9, 17, 19, 26). In this study ISCOM-matrices formulated with a QB saponin fraction from *Q. brasiliensis* (IMXQB) were tested as adjuvants for a seasonal influenza vaccine. As expected, the IMXQB obtained were cage-like structures, homogeneously distributed in size (average hydrostatic diameter of 40 nm) and negatively charged (16). IMXQB showed no hemolytic nor cytotoxic activities, as demonstrated by a hemolysis assay in rabbit RBCs and cytotoxicity assays in MDCK cells, respectively, confirming their safety (11).

Subcutaneous vaccination with TIV-IMXQB elicited higher IgM and IgG antibody levels than those generated by the unadjuvanted commercial TIV, and IgG titers were boosted after the second dose. We confirmed that our formulation, TIV-IMXQB, significantly enhanced IgG, IgG1, IgG2b, IgG3, and IgG2a antibody responses against the viral antigen after the booster vaccination compared with TIV alone, consistent with results previously reported by our group with other viral antigen (9, 22, 26). Importantly, immunization of animals with the TIV-IMXQB-adjuvanted vaccine elicited high titers of antibodies, as detected by both HAI and MN assays. Indeed, neutralizing antibodies are important as they block virus binding and entry into the respiratory cells. In addition, broadly neutralizing antibodies show a breadth of cross-reactivity, enabling them to neutralize many different strains within a subtype or group, or even between different types of influenza virus (35). The neutralizing antibody response may explain, in part, the lack of body weight loss in mice vaccinated with TIV-IMXQB and challenged with a lethal dose of A/Uruguay/897/2018 (H1N1)pdm09-like virus compared with the animals that received TIV alone, in addition to the almost complete clearance of the challenge virus in the lungs of mice (only one animal out of five had a very low but detectable virus titer). Of note, lung viral titers in animals immunized with the commercial TIV did not show significant differences with the mock groups, highlighting the efficacy of our investigational adjuvant, IMXQB.

The mucosal surfaces are the point of entry for many pathogens, including influenza and other respiratory viruses. One of the biggest challenges in combating these intracellular infections is to design safe vaccines that promote long-lasting induction of systemic and mucosal immunity, induce antigen-specific antibodies, and elicit robust T-cell immunity. We found that TIV-IMXQB but not TIV was able to prompt a positive DTH reaction. Indeed, DTH (which usually requires 12–24 hours for signs of inflammation to occur locally) is a common immune response that occurs through the direct action of sensitized T cells when stimulated by contact with the antigen. Specifically, DTH reaction has been linked to memory Th1 CD4^+^ T cells (36, 37), and our results suggest that a saponin-based IMXQB adjuvant promoted a T-cell response that included the differentiation to a Th1 phenotype, something that was not observed in the case of the non-adjuvanted TIV formulation. Animals inoculated with saponin-based nanoparticles also elicited more influenza IgG-ASCs than those inoculated with TIV alone. Interestingly, when we characterized the isotypes of the immunoglobulins secreted by these antibody-producing cells, we found that the TIV-IMXQB nanoparticle formulation induced significantly more influenza-specific IgG2a-ASCs than TIV alone. The TIV-IMXQB does not differ with TIV in the production of specific IgG1-ASC. The IgG2a isotype is a marker of polarization toward the Th1 response (9), which activates the classical pathway of complement and mediates antibody-dependent cell-mediated cytotoxicity, which inducing the efficient generation of cytotoxic T cells, promotes virus clearance and recovery after viral infection (2, 38). Our results show that TIV-IMXQB induced higher levels of Th1 (IFN-γ and TNF-α) and Th2 (IL-4 and IL-5) cytokines. In mice, Th1 immune responses are usually associated with enhanced isotype switching to IgG2a and IgG3 (promoted by INF-γ), which is effective for protection against intracellular infections, whereas Th2 responses promote the production of IgG1 (promoted by IL-4), which is required for protective immunity against extracellular infections and characterized by the production of IL-4, IL-5, IL-6, and IL-10 cytokines and IgG1 (37, 39). Furthermore, IgG2a is the predominant protective neutralizing antibody in experimental viral infections and provides protective immunity against lethal challenges with highly pathogenic and pandemic influenza strains in murine models (40, 41). It has been known for a long time that a bias toward the Th1-type response as can be induced by inoculation with *Quillaja* saponin preparations, improves the ability of an adjuvant to stimulate the production of IFN-γ-producing CD8^+^ T cells (7, 10, 12, 17, 42). Th1-mediated immunity induced by saponin adjuvants effectively destroys cancer or virally infected cells and may also cause tissue damage. This response is followed by a Th2 response, which is a repairing type of immune response and works by regulating the inflammatory process promoted by the adjuvant (13, 20). Likewise, it has been described that spherical nanoparticles in the size range between 20 and 100 nm, such as those used in this study, when administered systemically, induce a Th1-biased immune response (43, 44). Our results suggest that the adjuvanted vaccine (TIV-IMXQB) elicited a balanced Th1/Th2 response, which provides an advantage to its general use since the immune response represented by the two types of helper T cells is not skewed toward a particular profile (Th1 or Th2). Therefore, this adjuvant would be effective against intracellular pathogens (Th1 response) as well as extracellular pathogens (Th2 response) (20, 45).

On the other hand, analysis of spleen cell populations showed that the TIV-IMXQB vaccine promoted increased recruitment of effector CD4^+^ and CD8^+^ T cell phenotypes (CD62L^–^ CD127^–^) compared with TIV. The effector CD4^+^ and CD8^+^ T cells generated after the booster immunization are expected to include influenza-specific T cells that could provide a rapid response to clear the pathogen. By secreting cytokines and helping the B cells to produce antibodies, these cells orchestrate immune responses against a wide variety of microorganisms, adjusting the magnitude and persistence of responses (46).

Currently, most vaccines are administered intramuscularly, but alternative administration routes have been widely explored, particularly for mucosal pathogens such as influenza viruses. Nanoparticle vaccine delivery to mucosal tissues, for example in the nose, is a good alternative to promote protection against a specific pathogen (44). Intranasal vaccination is an efficient route to elicit robust humoral immune responses to weak immunogenic antigens. Recently, we reported that i.n. delivery of QB saponin-based ISCOMs co-formulated with influenza antigens induced higher titers of Ig2a, IgA, and HAI in mice than the commercial influenza vaccine (9). The findings of our study support those previous results. We found that SBA also promotes higher titers of protective neutralizing antibodies than TIV and that all mice vaccinated intranasally with TIV-IMXQB survived a lethal challenge with influenza virus, showing complete clearance of the virus in the lower respiratory tract, whereas animals receiving TIV showed a high lung viral load, and 75% reached the endpoint between days 6 and 7. The markedly weaker response promoted by intranasal TIV may be due to the degradation of the antigen prior to its processing by phagocytes. One of the current challenges in the design of effective i.n. vaccine delivery is to prevent the degradation of the antigen before it reaches the lymphoid organs. In fact, the success of this inoculation route is related to the protection of the antigen and the consequent successful induction of immunity in the mucosa. Some antigens may be poor immunogens or be inefficient in reaching the nasal mucosa upon intranasal vaccination; therefore, they may be degraded or not able to arrive in their conformational form at the lymphoid tissue, leading to the development of an ineffective immune response. Poor immunogenicity is usually solved with the addition of adjuvants (43), such as nanoparticles, which protect proteins from extracellular degradation and prolong their circulation in the immune system. This facilitates a more efficient cellular uptake by antigen-presenting cells and makes them capable of inducing potent antigen-specific humoral and cellular responses by promoting a higher level of cross-presentation (47). Here, we used a TIV-IMXQB, vaccine, delivered intranasally, and found that this conferred stronger protection against the antigen than the unadjuvanted commercial vaccine and triggered a robust immune response that protected animals against lethal challenge.

SBAs have been shown to be safe and potent adjuvants against viral infections. Recently, two vaccines have been licensed, one against malaria (Mosquirix™) and one against herpes zoster (Shingrix™) (8). Both vaccines are formulated with an AS01 adjuvant system containing monophosphoryl lipid A (MPL) and QS-21 saponin from *Q. saponaria* formulated in liposomes. Novavax, Inc. has recently announced that the Matrix-M-adjuvanted quadrivalent nanoparticle influenza vaccine was immunologically comparable to the licensed quadrivalent inactivated influenza vaccine for older adults (34). In the last decade, we have reported experimental evidence of chemical similarities (12, 21) and adjuvant activity of saponins belonging to the Quillajaceae family when they are co-formulated with a variety of viral antigens (9, 12, 20, 22, 23, 26, 31). The potential of QB saponins to offer alternative sources of QS-21 for use is clearly due to recent reports from our team (12, 21) and others (24, 48).

In summary, we have demonstrated the safety and increased ability of QB saponin-based nanoparticulate adjuvants to promote a balanced Th1/Th2 response, high levels of neutralizing antibodies, and improved HAI capacity in addition to the increased generation of effector CD4^+^ and CD8^+^ T cells. Consistent with that, we found that animals vaccinated with TIV-IMXQB achieved fast virus clearance when challenged with a lethal dose of A/Uruguay/897/2018 (H1N1)pdm09-like virus.

## 5 Conclusion

This study demonstrated that IMXQB nanosized particles formulated with QB saponin are safe and strong adjuvants for vaccines. The IMXQB adjuvant improved the potency of the commercial influenza vaccine by promoting a Th1/Th2 mixed phenotype. In addition, the animals inoculated subcutaneously or intranasally with the TIV adjuvanted with nanoparticles of QB and challenged with a lethal dose of A/Uruguay/897/2018 (H1N1)pdm09-like virus did not experience morbidity or mortality. In particular, the delivery of an unadjuvanted vaccine was much less effective in protecting animals than the IMXQB-adjuvanted vaccine, as most animals succumbed after the challenge. The QB saponin-based adjuvant (IMXQB) could be considered for use in influenza virus and other intracellular pathogen vaccines as an alternative to commercial *Q. saponaria* saponins.

## Data availability statement

The original contributions presented in the study are included in the article/supplementary material. Further inquiries can be directed to the corresponding authors.

## Ethics statement

The animal study was reviewed and approved according to the guidelines of the Comisión Honoraria de Experimentación Animal (CHEA-Universidad de la República) and was approved by the Universidad de la República Research Ethics Committee (approval number, N° 070153–000310–17).

## Author contributions

FS and MB designed the experiments. FS, MR-P, ND, SS, MM, and MB performed the experiments and analyzed or interpreted the data. FS, SC, and MB wrote the first draft of the manuscript. All authors were involved in critically revising the manuscript for important intellectual content. All authors contributed to the article and approved the submitted version.

## References

[1] Krammer F. SARS-CoV-2 vaccines in development. Nature (2020) 586:516–27. doi: 10.1038/s41586-020-2798-3 32967006

[2] Kim YH Hong KJ Kim H Nam JH. Influenza vaccines: past, present, and future. Rev Med Virol (2022) 32. doi: 10.1002/rmv.2243 PMC8209895 33949021

[3] Tregoning JS Russell RF Kinnear E. Adjuvanted influenza vaccines. Hum Vaccines Immunother (2018) 14:550–64. doi: 10.1080/21645515.2017.1415684 PMC5861793 29232151

[4] DiazGranados CA Dunning AJ Jordanov E Landolfi V Denis M Talbot HK. High-dose trivalent influenza vaccine compared to standard dose vaccine in elderly adults: safety, immunogenicity and relative efficacy during the 2009-2010 season. Vaccine (2013) 31:861–6. doi: 10.1016/j.vaccine.2012.12.013 23261045

[5] Pulendran B S Arunachalam P O’Hagan DT. Emerging concepts in the science of vaccine adjuvants. Nat Rev Drug Discovery (2021), 1–22. doi: 10.1038/s41573-021-00163-y 33824489 PMC8023785

[6] Giudice G Del, Rappuoli R. Inactivated and adjuvanted influenza vaccines. Curr Top Microbiol Immunol (2015) 386:151–80. doi: 10.1007/82_2014_406 25038938

[7] Sun HX Xie Y Ye YP. Advances in saponin-based adjuvants. Vaccine (2009) 27:1787–96. doi: 10.1016/j.vaccine.2009.01.091 19208455

[8] Lacaille-Dubois M-D. Updated insights into the mechanism of action and clinical profile of the immunoadjuvant QS-21: a review. Phytomedicine (2019) 60:152905. doi: 10.1016/j.phymed.2019.152905 31182297 PMC7127804

[9] Rivera-patron M Baz M Roehe PM Cibulski SP Silveira F. ISCOM-like nanoparticles formulated with quillaja brasiliensis saponins are promising adjuvants for seasonal influenza vaccines. (2021). pp. 1–18. doi: 10.3390/vaccines9111350. PMC8621233 34835281

[10] Zhu D Tuo W. QS-21: a potent vaccine adjuvant. Nat Prod Chem Res (2016) 3:3–4. doi: 10.4172/2329-6836.1000e113 PMC4874334 27213168

[11] Cibulski SP Mourglia-Ettlin G Teixeira TF Quirici L Roehe PM Ferreira F. Novel ISCOMs from quillaja brasiliensis saponins induce mucosal and systemic antibody production, T-cell responses and improved antigen uptake. Vaccine (2016) 34:1162–71. doi: 10.1016/j.vaccine.2016.01.029 26826546

[12] Cibulski S Rivera-Patron M Suárez N Pirez M Rossi S Yendo AC. Leaf saponins of quillaja brasiliensis enhance long-term specific immune responses and promote dose-sparing effect in BVDV experimental vaccines. Vaccine (2018) 36:55–65. doi: 10.1016/j.vaccine.2017.11.030 29174676

[13] Marciani DJ. Elucidating the mechanisms of action of saponin-derived adjuvants. Trends Pharmacol Sci (2018) xx:1–13. doi: 10.1016/j.tips.2018.03.005 29655658

[14] Didierlaurent AM Laupeze B Di Pasquale A Hergli N Collignon C Garcon N. Adjuvant system AS01: helping to overcome the challenges of modern vaccines. Expert Rev Vaccines (2016) 0584:1–9. doi: 10.1080/14760584.2016.1213632 27448771

[15] Morein B Sundquist B Höglund S Dalsgaard K Osterhaus A. Iscom, a novel structure for antigenic presentation of membrane proteins from enveloped viruses. Nature (1984) 308:457–60. doi: 10.1038/308457a0 6709052

[16] Rivera-Patron M Cibulski SP Miraballes I Silveira F. “Formulation of IMXQB: nanoparticles based on quillaja brasiliensis saponins to be used as vaccine adjuvants.,”. In: Fett-neto AG, editor. Methods in molecular biology (Clifton, N.J.). Porto Alegre, Brazi: Methods Mol Biol (2022). p. 183–91. doi: 10.1007/978-1-0716-2185-1_15 35508839

[17] Morelli AB Maraskovsky E. “ISCOMATRIX adjuvant in the development of prophylactic and therapeutic vaccines.,”. In: Schijns VEJC O’Hagan DT, editors. Immunopotentiators in modern vaccines. London (2016). p. 311–32.

[18] Bigaeva E van Doorn E Liu H Hak E. Meta-analysis on randomized controlled trials of vaccines with QS-21 or ISCOMATRIX adjuvant: safety and tolerability. PloS One (2016) 11:e0154757. doi: 10.1371/journal.pone.0154757 27149269 PMC4858302

[19] McKenzie A Watt M Gittleson C. ISCOMATRIX vaccines: safety in human clinical studies. Hum Vaccin (2010) 6:237–46. doi: 10.4161/hv.6.3.10754 20595811

[20] Magedans YVS Yendo ACA De CF Gosmann G Arthur G. Foamy matters: an update on quillaja saponins and their use as immunoadjuvants. Future Med Chem (2019). doi: 10.4155/fmc-2018-0438 31304830

[21] Cibulski S Amorim T Joanda DS Raimundo P Mangueira Y Silva L. ISCOM − matrices nanoformulation using the raw aqueous extract of quillaja lancifolia ( q . brasiliensis ). Bionanoscience (2022). doi: 10.1007/s12668-022-01023-8 PMC9362619 35967762

[22] Cibulski S Varela APM Teixeira TF Cancela MP Sesterheim P Souza DO. Zika virus envelope domain III recombinant protein delivered with saponin-based nanoadjuvant from quillaja brasiliensis enhances anti-zika immune responses, including neutralizing antibodies and splenocyte proliferation. Front Immunol (2021) 12:632714. doi: 10.3389/fimmu.2021.632714 33746970 PMC7969523

[23] Silveira F Cibulski SP Varela AP Marqués JM Chabalgoity A de Costa F. Quillaja brasiliensis saponins are less toxic than quil a and have similar properties when used as an adjuvant for a viral antigen preparation. Vaccine (2011) 29:9177–82. doi: 10.1016/j.vaccine.2011.09.137 22001878

[24] Wallace F Fontana C Ferreira F. Structure elucidation of triterpenoid saponins found in an immunoadjuvant preparation of quillaja brasiliensis using. Molecules (2022), 1–13. doi: 10.3390/molecules27082402 35458600 PMC9024837

[25] Cunningham AL Lal H Kovac M Chlibek R Hwang S-J Díez-Domingo J. Efficacy of the herpes zoster subunit vaccine in adults 70 years of age or older. N Engl J Med (2016) 375:1019–32. doi: 10.1056/nejmoa1603800 27626517

[26] Cibulski S Teixeira TF Varela APM de Lima MF Casanova G Nascimento YM. IMXQB-80: a quillaja brasiliensis saponin-based nanoadjuvant enhances zika virus specific immune responses in mice. Vaccine (2020) 39:571–9. doi: 10.1016/j.vaccine.2020.12.004 33339669

[27] Yendo A de Costa F Kauffmann C Fleck J Gosmann G Fett-Neto A. “Purification of an immunoadjuvant saponin fraction from quillaja brasiliensis leaves by reversed-phase silica gel chromatography.,”. In: Fox CB, editor. Methods in molecular biology. New York: Springer (2017). p. 87–93. doi: 10.1007/978-1-4939-6445-1_6 27718187

[28] Silveira F Rossi S Fernández C Gosmann G Schenkel E Ferreira F. Alum - type adjuvant effect of non - haemolytic saponins puri fi ed from ilex and passi fl ora spp. Phyther Res (2011) 1788:1783–8. doi: 10.1002/ptr.3454 21480409

[29] Webster R Cox N Stohr K. “WHO manual on animal influenza diagnosis and surveillance.,”. In: WHO/CDS/CDR/2002.5 rev. 1 (2002). p. 105. Available at: http://scholar.google.com/scholar?hl=en&btnG=Search&q=intitle:WHO+Manual+on+Animal+Influenza+Diagnosis+and+Surveillance+World+Health+Organization+Department+of+Communicable+Disease+Surveillance+and#1.

[30] Baz M Boonnak K Paskel M Santos C Powell T Townsend A. Nonreplicating influenza a virus vaccines confer broad protection against lethal challenge. MBio (2015) 6. doi: 10.1128/mBio.01487-15 PMC4620468 26489862

[31] Cibulski SP Silveira F Mourglia-Ettlin G Teixeira TF dos Santos HF Yendo AC. Quillaja brasiliensis saponins induce robust humoral and cellular responses in a bovine viral diarrhea virus vaccine in mice. Comp Immunol Microbiol Infect Dis (2016) 45:1–8. doi: 10.1016/j.cimid.2016.01.004 27012913

[32] Baz M Paskel M Matsuoka Y Zengel JR Cheng X Treanor JJ. A single dose of an avian H3N8 influenza virus vaccine is highly immunogenic and efficacious against a recently emerged seal influenza virus in mice and ferrets. J Virol (2015) 89:6907–17. doi: 10.1128/JVI.00280-15 PMC4468475 25903333

[33] Baz M Paskel M Matsuoka Y Zengel J Cheng X Treanor JJ. A live attenuated equine H3N8 influenza vaccine is highly immunogenic and efficacious in mice and ferrets. J Virol (2015) 89:1652–9. doi: 10.1128/JVI.02449-14 PMC4300743 25410860

[34] Shinde V Cho I Plested JS Agrawal S Fiske J Cai R. Comparison of the safety and immunogenicity of a novel matrix-m-adjuvanted nanoparticle influenza vaccine with a quadrivalent seasonal influenza vaccine in older adults: a phase 3 randomised controlled trial. Lancet Infect Dis (2022) 22:73–84. doi: 10.1016/S1473-3099(21)00192-4 34563277

[35] Laursen NS Wilson IA. Broadly neutralizing antibodies against influenza viruses. Antiviral Res (2013) 98:476–83. doi: 10.1016/j.antiviral.2013.03.021 PMC3987986 23583287

[36] Cher DJ Mosmann TR. Two types of murine helper T cell clone. II. delayed-type hypersensitivity is mediated by TH1 clones. J Imunol (1987) 138:3688–94. doi: 10.4049/jimmunol.138.11.3688 2953788

[37] Romagnani S. Type 1 T helper and type 2 T helper cells: functions, regulation and role in protection and disease. Int J Clin Lab Res (1991) 21:152–8. doi: 10.1007/BF02591635 1687725

[38] Principi N Esposito S. Adjuvanted influenza vaccines. Hum Vaccin Immunother (2012) 8:59–66. doi: 10.4161/hv.8.1.18011 22252004

[39] O’Garra A. Cytokines induce the development of functionally heterogeneous T helper cell subsets. Immunity (1998) 8:275–83. doi: 10.1016/S1074-7613(00)80533-6 9529145

[40] Liang H Tang J Liu Z Liu Y Huang Y Xu Y. ZIKV infection induces robust Th1-like tfh cell and long-term protective antibody responses in immunocompetent mice. Nat Commun (2019) 10:1–16. doi: 10.1038/s41467-019-11754-0 31455769 PMC6712032

[41] Miyauchi K Sugimoto-Ishige A Harada Y Adachi Y Usami Y Kaji T. Protective neutralizing influenza antibody response in the absence of T follicular helper cells. Nat Immunol (2016) 17:1447–58. doi: 10.1038/ni.3563 27798619

[42] Wilson NS Yang B Morelli AB Koernig S Yang A Loeser S. ISCOMATRIX vaccines mediate CD8 T-cell cross-priming by a MyD88-dependent signaling pathway. Immunol Cell Biol (2012) 90:540–52. doi: 10.1038/icb.2011.71 PMC3365289 21894173

[43] Thakur A Foged C. Nanoparticles for mucosal vaccine delivery. Elsevier Ltd (2020), 603–46. doi: 10.1016/B978-0-08-102985-5.00025-5

[44] van Riet E Ainai A Suzuki T Kersten G Hasegawa H. Combatting infectious diseases; nanotechnology as a platform for rational vaccine design. Adv Drug Delivery Rev (2014) 74:28–34. doi: 10.1016/j.addr.2014.05.011 24862579

[45] Marciani DJ Pathak AK Reynolds RC Seitz L May RD. Altered immunomodulating and toxicological properties of degraded quillaja saponaria Molina saponins. Int Immunopharmacol (2001) 1:813–8. doi: 10.1016/S1567-5769(01)00016-9 11357894

[46] Bugya Z Prechl J Szénási T Nemes É Bácsi A Koncz G. Multiple levels of immunological memory and their association with vaccination. Vaccines (2021) 9:1–25. doi: 10.3390/vaccines9020174 PMC7922266 33669597

[47] Taki A Smooker P. Small wonders–the use of nanoparticles for delivering antigen. Vaccines (2015) 3:638–61. doi: 10.3390/vaccines3030638 PMC4586471 26350599

[48] Wallace F Bennadji Z Ferreira F Olivaro C. Analysis of an immunoadjuvant saponin fraction from quillaja brasiliensis leaves by electrospray ionization ion trap multiple-stage mass spectrometry. Phytochem Lett (2017) 20:228–33. doi: 10.1016/j.phytol.2017.04.020

